# Immunization delivery in the second year of life in Ghana: the need for a multi-faceted approach

**DOI:** 10.11604/pamj.supp.2017.27.3.12182

**Published:** 2017-06-21

**Authors:** Mawuli Nyaku, Melissa Wardle, Jodi Vanden Eng, Lynnette Ametewee, George Bonsu, Joseph Kwadwo Larbi Opare, Laura Conklin

**Affiliations:** 1 Centers for Disease Control and Prevention, Center for Global Health, Global Immunization Division, 1600 Clifton Road NE, Mailstop A-04, Atlanta GA 30329, USA; 2 Ghana Health Service, Public Health Division, Disease Control and Prevention Department, Expanded Programme on Immunization, Korle Bu, Accra, Ghana; 3 African Field Epidemiology Network, Lugogo House, Plot 42 Lugogo By Pass, Kampala, Uganda

**Keywords:** Ghana, immunization, second year of life, measles-containing-vaccine, dropout rate, defaulter tracing

## Abstract

**Introduction:**

in 2012, pneumococcal conjugate vaccine (PCV), rotavirus vaccine and a second dose of measles-containing vaccine (MCV2) were introduced into the Expanded Program on Immunization (EPI) in Ghana. According to Ghana’s EPI schedule, PCV and rotavirus vaccine are given in the first year of life and MCV2 in the second year of life (2YL) at 18 months. Although coverage with the last doses of PCV and rotavirus vaccine reached almost 90% coverage within four years of introduction, MCV2 coverage did not rise above 70%. The World Health Organization Global Measles and Rubella Strategic Plan established a 2020 milestone to achieve at least 95% coverage with the first and second doses of measles-containing vaccine in each district and nationally. We developed a project to address challenges to delivery of immunizations and other child health services at the 18-month visit and throughout the 2YL.

**Methods:**

from March to April 2016, we conducted a cluster survey of households (HHs) with children 24-35 months of age in three regions in Ghana to assess knowledge, attitudes and beliefs among caregivers about immunization during the 2YL and to collect childhood vaccination history data using vaccination cards. Three independent samples were selected from the Northern (NR), Volta (VR), and Greater Accra (GAR) regions. A survey and direct observations were performed a ta representative sample of health facilities (HFs) providing immunization services in the same regions to further characterize barriers to immunization access, utilization and delivery in the 2YL.

**Results:**

data on a total of 464 children ages 24-35 months were collected in the HH survey: 211 in NR, 153 in VR, and 100 in GAR (response rate > 99%). First dose of measles-containing vaccine (MCV1) coverage was (NR: 87%, VR: 96%, GAR: 99%); however, MCV2 coverage was lower (NR: 60%, VR: 83%, GAR: 70%). MCV1 to MCV2 dropout was 32% in NR, 14% in VR, and 31% in GAR. Caregiver awareness of immunization against measles was 69% in NR, 75% in VR, and 68% in GAR yet less than half knew the recommended ages for receiving the vaccine, (NR: 4%, VR: 9%, GAR: 44%). Among 160 HFs participating in the survey (>50 in each region), most lacked a defaulter tracing system (NR: 94%,VR: 76%,GAR: 85%). A varying proportion of HCWs correctly indicated how to record a catch-up first dose of MCV administered to an 18-month-old child in the 12-23 month immunization register (NR: 38%, VR: 55%, GAR: 67%) and on the vaccination card (NR: 54%, VR: 53%, GAR: 76%). Although more than half of caregivers would accept text messages, (NR: 57%, VR: 78%, GAR: 96%) including reminders, related to their child’s immunizations, < 10% HFs were utilizing this practice.

**Conclusion:**

challenges encountered with the establishment of an immunization visit beyond the first year of life included knowledge gaps among caregivers, high dropout rates between MCV1 and MCV2 in all study regions, and a lack of defaulter tracing systems in most healthcare facilities providing childhood immunizations. Targeted strategies that promote behavioral, cultural, and policy changes are needed to strengthen 2YL child health service delivery and improve vaccination coverage.

## Introduction

Child health services provided during the second year of life (2YL) provide opportunities to administer new vaccines recommended for older children, booster doses of existing vaccines and other child health interventions. In Ghana, an 18-month well-child visit was first established to provide vitamin A supplementation, growth monitoring, deworming, and long-lasting insecticide-treated mosquito bednet distribution [1]. As the country moved towards implementing the World Health Organization (WHO) Global Measles and Rubella Strategic Plan and achieving the plan’s 2020 milestone of 95% district and national coverage with two doses of measles-containing vaccine (MCV), introduction of a 2YL service delivery platform provided a mechanism for offering a second MCV dose [2]. At the same time, it would serve to facilitate introduction of serogroup A meningococcal conjugate vaccine (MenA) and improve coverage of vaccines scheduled for the first year of life through catch-up immunizations.

Ghana has had relatively high vaccination coverage (≥85%) for infant antigens including MCV1 since 2007 [3]. The country has been a leader for vaccine introduction in the African Region, being one of the first to introduce the pentavalent (diphtheria-pertussis-tetanus-hepatitis B-Haemophilus influenzae type b) vaccine in 2002, and pneumococcal conjugate vaccine (PCV) and rotavirus vaccine simultaneously in 2012. Ghana also introduced MCV2, the first non-infant vaccine in the childhood immunization schedule, in 2012 as a newly established 18-month visit. At the time of MCV2 introduction, Ghana’s Expanded Program on Immunization (EPI) conducted planning activities including: establishing a national immunization sub-committee for training, logistics and social mobilization; conducting regional, district, and health worker training and capacity building activities; and coordinating social mobilization and demand generation activities at the community levels. Despite those efforts, MCV2 coverage remained below 70% four years after introduction while coverage with the last doses of PCV and rotavirus vaccine had reached ≥ 89% during the same period [3]. Both PCV and rotavirus vaccine are administered during established visits for pentavalent vaccine.

As part of the Global Health Security Agenda’s objectives of strengthening countries’ capacities to prevent, detect, and respond to infectious disease threats, the U.S. Centers for Disease Control and Prevention (CDC) is collaborating with the Ghana Health Service (GHS) to identify and address health facility and community level barriers contributing to low MCV2 coverage and to strengthen a 2YL service delivery platform to address those barriers [4]. A major intended outcome of the project is to provide a model for other countries considering expanding delivery of immunizations and other child health services during the 2YL. This paper presents findings from a baseline survey of households and health facilities conducted in March 2016 in three regions in Ghana, addressing vaccination coverage indicators; awareness, knowledge, attitudes and beliefs among caregivers; and healthcare worker knowledge and practices; to inform the project’s multi-faceted approach to strengthening Ghana’s 2YL service delivery platform.

## Methods

### Study site and survey design

Ghana has an under-5-year-old population of approximately 4 million within a total population of approximately 27 million [5]. There are 10 administrative regions and over 3,000 health facilities that provide immunization services to children. We selected three regions –Northern Region (NR), Volta Region (VR) and Greater Accra Region (GAR) – for our activities based on high numbers of districts with low MCV2 coverage and inequities in access to immunization services. NR is the largest region in Ghana, with a low population density of 91 persons/square mile (sq. mi.) and great distances between health facilities. VR has a population density of 270 persons/sq. mi. and is separated from the rest of the country by Ghana’s largest lake, Lake Volta. GAR is the smallest of the administrative regions in Ghana, has the highest population density of 3,200 persons/sq. mi. and large migrant and transient populations. In March 2016, we conducted a community-based cross-sectional household survey and a health facility survey and needs assessment in each region. The baseline survey included children ages 12-23 months and ages 24-35 months; however, only data on children 24-35 months are presented here.

### Household survey

Sample sizes were calculated to measure a difference of 15% in MCV2 coverage among children 24-35 months of age from baseline to follow-up survey, with 80% power at a significance level of alpha = 0.05 using a one-sided test and adjusting for 10% non-response, the proportion of households with eligible children, and intra-class correlation. Sample sizes were generated separately by region where baseline proportions, intra-class cluster correlation, and cluster sizes were approximated using the 2014 Ghana Demographic Health Survey (DHS) results [6].

The household survey used a two-stage sample design. The first stage selection was done by the DHS which selected 37 enumeration areas in NR, 41 in the VR, and 48 in GAR and provided the corresponding selection probabilities. The second stage involved selecting households by simple random sampling from the DHS listings. A total of 50 (NR), 65 (VR), and 60 (GAR) households were selected in each enumeration area. A standardized questionnaire was administered by survey teams. If a household had more than one eligible participant 24-35 months of age, one child was randomly selected as the subject for the interview. Eligibility criteria included that the child lived in Ghana from age 12 months to age 23 months, and the child’s caregiver was older than 16 years of age and provided consent at the time of the interview. Questions covered family characteristics and demographics; immunization awareness, knowledge, attitudes and beliefs; and childhood vaccination history using vaccination card data.

### Health facility

Health facilities were selected using systematic sampling from a master list of health facilities offering immunization services provided by Ghana’s EPI. We calculated that a minimum of 56 health facilities should be sampled per region to provide 80% power to detect a 20% to 45% difference in the proportion offering catch-up services from baseline to follow-up survey, assuming 10% non-response.

The health facility questionnaire consisted of three modules. The first module was administered to all health care workers (HCWs) that provided immunizations and were available for interview, and covered general facility staffing information and catchment population; availability, quality and management of immunization services and materials; immunization sessions and scheduling; training and supervision; and child registration and tracking. The second module was administered to a maximum of two eligible HCWs at each health facility and covered information about staff knowledge, attitudes, and beliefs. If there were more than two eligible HCWs, only two were selected for interviewing using simple random sampling. The third module was a detailed review of the immunization registers, tally sheets, and monthly reporting forms at selected health facilities.

### Data management and analyses

Data were entered directly into Android smartphones (BLU Studio 5.5 S, Doral, FL, USA, Android version 4.2.2) using questionnaires developed with Open Data Kit software (opendatakit.org, Seattle, WA, USA) and uploaded to a secure cloud server [7]. Health facility third module data were collected using paper tools and later entered into Epi Info™ version 7 [8]. Data were downloaded into Microsoft^®^ Excel databases and transferred to SAS version 9.3 (SAS Institute, Cary, NC) for data management and analysis. Data validation was performed by identifying unlikely questionnaire start and end times, and by checking GPS coordinates using ArcGIS software; data identified as false data entry were removed. Specialized analysis survey procedures in SAS (Proc Survey) were used to produce valid estimates and calculate standard errors to account for clustering at the primary sampling unit (district) level and sampling weights. Descriptive analyses and weighted proportions are presented.

Vaccination indicators including coverage of pentavalent vaccine, MCV1 and MCV2, were estimated for children with vaccination cards available during the time of the survey. A child was defined as fully vaccinated if they had received all antigens recommended by GHS by age 24 months (1 dose of BCG, 4 doses of OPV, 3 doses of pentavalent vaccine, 3 doses of PCV, 2 doses of rotavirus vaccine, 2 doses of MCV, and 1 dose of yellow fever vaccine). Analyses were done to compare dropout between selected immunizations given in the first year of life (first dose of pentavalent vaccine and MCV1) and between doses given in the first and second years of life (MCV1 and MCV2).

### Ethical approval

This project was determined to be non-research by the CDC Human Subjects Office. Approval for the project was obtained from the Ghana Health Service’s ethics review board.

## Results

### Household survey

#### Summary of sample

Overall, 7,330 households from NR, VR, and GAR were sampled for children 24-35 months of age. In NR, 1850 households were sampled; 342 were not located, 990 were not eligible, 56 were excluded due to false data entry, and 2 did not provide consent. Of the 460 completed interviews in NR, 211 households had a child 24-35 months of age. In VR, 2,665 households were sampled; 458 were not located, 1,786 were not eligible, and 109 were excluded due to false data entry. Of the 312 completed interviews in VR, 153 households had a child 24-35 months of age. In GAR, 2,880 households were sampled. Of the selected households 773 were not located, 1,477 were not eligible, 406 were excluded due to false data entry, and 1 did not provide consent. Of the 223 completed interviews in GAR, 100 households had a child 24-35 months of age.

#### Background characteristics of household, child, and caregivers

Background characteristics for the surveyed households can be found in Table 1. In all regions, most respondents indicated living at the same location for more than a year, were married mothers with a mean age range of 29-31 years, and lived in households with average size of 5-7 inhabitants. There were differences among regions in the following characteristics: proportion of surveyed children who were female (NR: 32%,VR: 50%, GAR: 49%); proportion of children first born (NR: 20%, VR: 21%, GAR: 41%); proportion of mothers who never attended school (NR: 73%, VR: 31%, GAR: 10%). In NR the majority (52%) of mothers were Muslim while in VR and GAR the majority (87%-89%) were Christian.

**Table 1:** household and maternal characteristics of children 24-35 months in three regions in Ghana, baseline survey, March 2016

	Northern RegionN = 211	Volta RegionN = 153	Greater Accra RegionN = 100
	Number of responses (%)^+^	Number of responses (%)^+^	Number of responses (%)^+^
**Relationship of respondent to child**			
Mother	191 (93)	127 (84)	88 (80)
Father	14 (5)	11 (7)	6 (5)
Other	6 (2)	15 (9)	7 (15)
**Length of time at current residence**			
1 year or longer	203 (96)	148 (96)	99 (99)
Less than 1 year	7 (4)	5 (4)	1 (1)
**Mean number of people per household**	7	6	5
**Sex of child**			
female	86 (32)	78 (50)	50 (49)
**Birth order of child**			
1^st^ born	44 (20)	33 (21)	34 (41)
2^nd^ born or later	167 (80)	120 (79)	66 (59)
**Mother's age (mean)**	29	30	31
**Mother's marital status**			
Married	205 (98)	143 (93)	85 (91)
**Mother's educational level**			
Never attended school	145 (73)	49 (31)	13 (10)
Primary	15 (6)	30 (18)	16 (24)
Secondary or higher	51 (21)	74 (52)	71 (66)
**Mother's religion**			
Christian	72 (33)	135 (87)	87 (89)
Muslim	119 (52)	6 (4)	9 (7)
Traditionalist	16 (12)	11 (8)	2 (3)
None	4 (2)	1 (1)	2 (1)
**Mother's occupation**			
Farmer/Laborer/Fisherman	108 (54)	51 (34)	2 (2)
Trader/Merchant	58 (24)	61 (39)	51 (51)
Unemployed	19 (10)	16 (11)	15 (12)
Other	26 (13)	25 (16)	32 (35)


+ percentages account for clustering at the primary sampling unit (district) level and sampling weights

#### Child vaccination coverage indicators

Vaccination coverage indicators stratified by region are shown in Table 2. Vaccination card availability was >80% for all regions. Coverage estimates of third-dose pentavalent vaccine and MCV1 were greater than 95% in VR and GAR regions while estimates in NR were 88% (95% CI: 80% – 94%) for third-dose pentavalent vaccine and 87% (95% CI: 78% – 92%) for MCV1. Coverage estimates for MCV2 were lower in each region: 60% (95% CI: 46% - 72%) in NR, 83% (95% CI: 74% - 90%) in VR, and 70% (95% CI: 49% – 85%) in GAR. The estimated proportion of children fully vaccinated was highest in VR at 77% (95% CI: 65%– 85%), followed by GAR at 67% (95% CI: 49% – 85%) and NR at 44% (95% CI: 35% – 54%).

**Table 2:** characteristics of healthcare workers (HCWs) participating in health facility survey and individual interviews in three regions in Ghana, baseline survey, March 2016

	Northern RegionN=110 HCWs	Volta RegionN=103 HCWs	Greater Accra RegionN=109 HCWs
	Number of responses (%)^++^	Number of responses (%)^++^	Number of responses (%)^++^
**Designation of participant**			
Community health nurse	70 (64)	60 (58)	50 (46)
In-Charge/Principal community health nurse	9 (8)	27 (26)	34 (31)
Senior community health nurse	4 (4)	10 (10)	19 (17)
Disease control officer	20 (18)	0 (0)	0 (0)
Other^+^	7 (6)	6 (6)	6 (6)
**Mean** number of HCWs who administer immunizations at health facility	2	3	3
**Mean** number of years participant working in immunization services	4	4	8
**Mean** number of years participant working in immunization services at current health facility	2	3	3
**Number of individual HCWs selected for one-on-one interviews**	**N = 72 HCWs**	**N = 85 HCWs**	**N = 83 HCWs**
**Immunization job duties**			
Administering immunizations	67 (89)	83 (99)	81 (96)
Health education/Counseling	53 (72)	69 (84)	69 (82)
Recording in tallying books	24 (36)	48 (58)	55 (65)
Registering children	32 (50)	47 (57)	46 (52)
Home visits	16 (21)	39 (50)	32 (37)
Writing monthly reports	18 (24)	33 (42)	33 (36)
Managing immunization stock	15 (21)	14 (17)	18 (22)
Supervising others who administer immunizations	3 (4)	6 (10)	6 (7)
Defaulter tracing	1 (1)	3 (2)	1 (1)
Cold chain	0 (0)	2 (2)	1 (1)
**Time since last formal EPI training**			
<1 year	14 (18)	13 (15)	34 (38)
>1 year	15 (23)	25 (28)	17 (26)
Don't know	43 (59)	47 (58)	32 (37)

+ Other includes: superintendent community health nurse, field technician, community mental health officer, nutrition officer, public health nurse

++ percentages account for clustering at the primary sampling unit (district) level and sampling weights

Dropout between first-dose pentavalent vaccine and MCV1 was slightly greater than 10% in NR (Table 2). However, dropout between MCV1 and MCV2 was >10% for all regions (NR: 32%, VR: 14%, GAR: 31%). Few vaccination cards had a recorded return date for MCV2 written in the allocated space (NR: 5%, VR:1%, GAR: 19%).

#### Caregiver knowledge, behaviors, and beliefs

Caregiver knowledge, behaviors, and beliefs data are shown in Table 2. More than two-thirds of caregivers were aware of measles immunization for their child, (NR: 69%, VR: 75%, GAR 68%), yet only 4% in NR, 9% in VR, and 44% in GAR indicated the correct recommended ages of measles immunization doses at 9 months and 18 months. Approximately half of all caregivers indicated it was equally important to vaccinate infants (<12 months) and older children (12-23 months) (NR: 42%, VR: 54%, GAR: 60%). More than three-quarters of caregivers in each region indicated that they took their child for child health services at 18 months, (NR: 75%, VR: 82%, GAR: 78%). Growth monitoring was the most common reason given (NR: 54%, VR: 48%, GAR: 62%), followed by measles immunization (NR: 14%, VR: 34%, GAR: 30%).

Healthcare providers were the most common and most trusted source of immunization information indicated by caregivers (NR: 56%, VR: 77%, GAR: 95%). In NR and VR, caregivers also indicated a traditional method of communication, the town crier who beats agong-gong, a metallic percussion instrument to draw people’s attention, as a common (19% and 26%, respectively) source of immunization information. Caregivers varied in receptivity to text messages as a form of communication about their child’s immunizations, including reminders for return visits (NR: 57%, VR: 78%, GAR: 96%).

Caregiver attitudes about receiving immunizations in school were assessed among a subset of caregivers who indicated their children stayed with another adult during the day for childcare. Among these children, the proportion that attended a formal daycare were 28% in NR, 47% in VR, and 94% in GAR. Of those, 87% of caregivers in NR, 100% in VR, and 87% in GAR were highly receptive to their child being immunized in school.

### Health facility survey

#### Characteristics of healthcare workers participating in HF survey

Of 168 health facilities selected to participate in the baseline survey, eight health facilities (4 in NR, 1 in VR, and 2 in GAR) did not participate. The numbers of participating health facilities were 52 in NR, 55 in VR, and 53 in GAR. Within those health facilities, a total of 110 HCWs from NR, 103 from VR, and 109 from GAR, with an average of two health workers per health facility, participated in first module of the health facility survey (Table 3). Various health worker designations were represented, with community health nurses and in-charge community health nurses making up more than 70% of participants. The average number of years working in immunization service delivery was higher in GAR (8 years) compared to NR and VR (4 years), while average number of years working at the current health facility was 2 years in NR and 3 years in VR and GAR.

**Table 3:** child immunization coverage indicators and caregiver knowledge, attitude, and behaviors related to immunizations in the second year of life among children 24-35 months in 3 regions in Ghana, baseline survey, March 2016

	Northern RegionN = 211		Volta RegionN = 153		Greater Accra RegionN = 100	
	Number of responses	% (95%CI)	Number of responses	% (95%CI)	Number of responses	% (95%CI)
**Vaccination card data**						
**Card available for review**	189	90 (83-94)	130	88 (80-92)	73	81 (69-90)
**Immunization coverage estimates among card holders**						
Third-dose pentavalent vaccine	168	88 (80-94)	126	96 (88-99)	72	95 (76-99)
MCV1	161	87 (78-92)	124	96 (90-99)	72	99 (94-100)
MCV2	113	60 (46-72)	106	83 (74-90)	54	70 (49-85)
**Fully immunized (among card holders)+**	88	44 (35-54)	98	77 (65-85)	51	67 (47-83)
**Immunization dropout rates**						
First-dose pentavalent vaccine to MCV1	20	11 (6-19)	5	4 (2-9)	0	0 (0-0)
MCV1 to MCV2	52	32 (23-44)	20	14 (8-24)	20	31 (16-52)
**Card indicated return date for MCV2**	12	5 (2-15)	3	1 (0-5)	7	19 (5-50)
**Caregiver knowledge, attitudes, and behaviors**						
**Aware of immunization against measles**	141	69 (55-80)	113	75 (64-84)	72	68 (51-81)
**Ages immunizations against measles routinely given to children**						
Both 9 & 18 months	9	4 (1-9)	10	9 (5-16)	23	44 (26-64)
9 months	30	16 (8-29)	42	35 (23-50)	20	29 (17-46)
18 months	4	2 (1-6)	9	9 (4-18)	3	3 (1-9)
Neither	35	27 (18-40)	14	12 (6-23)	11	12 (6-22)
Don't know	63	51 (38-64)	38	35 (23-50)	15	13 (6-27)
**Attended 18-month well-child visit**	153	75 (61-86)	125	82 (74-88)	77	78 (64-87)
**Main reasons for bringing child for 18-month well-child visit**						
Measles vaccine	26	14 (7-27)	41	34 (23-45)	27	30 (18-46)
Growth monitoring	70	54 (39-68)	59	48 (35-61)	40	62 (45-76)
Other^+^	57	32 (23-43)	25	19 (11-31)	10	8 (4-17)
**Importance of immunizations by age**						
Same importance	83	42 (30-54)	76	54 (41-66)	54	60 (43-75)
More important to vaccinate infants less than 12 months	111	51 (40-62)	69	41 (29-54)	39	36 (23-53)
More important to vaccinate children 12-23 months	2	1 (0-3)	5	4 (2-8)	2	1 (0-6)
Don’t know	14	7 (3-15)	3	2 (1-6)	3	2 (1-9)
**Commonly reported sources of immunization information**						
Healthcare provider	145	68 (52-80)	129	86 (73-94)	98	91 (74-97)
Family and Friends	55	29 (20-42)	34	20 (13-30)	10	11 (5-24)
Gong-gong++	40	19 (9-35)	35	26 (14-44)	2	1 (0-8)
**Most trusted source of immunization information**						
Healthcare provider	125	56 (45-66)	115	77 (64-86)	92	95 (89-98)
Gong-gong++	19	10 (4-26)	14	9 (3-24)	2	1 (0-6)
**Would be willing to receive text messages about child’s immunizations including reminders**	119	57 (44-68)	117	78 (68-86)	94	96 (89-98)
**Child stays with another adult during the day for childcare**	61	35 (23-49)	25	16 (8-30)	42	30 (17-48)
**Child attends daycare+++**	28	28 (12-52)	11	47 (21-74)	39	94 (82-98)
**Receptive to child being immunized at a daycare++++**	24	87 (63-96)	11	100 (0-0)	32	87 (73-94)

Abbreviations: first-dose measles-containing vaccine (MCV1); second-dose measles-containing vaccine (MCV2); diphtheria-tetanus-pertussis-hepatitis B-*Haemophilus influenzae* type b vaccine (first-dose pentavalent vaccine); diphtheria-tetanus-pertussis-hepatitis B-*Haemophilus influenzae* type b vaccine (second-dose pentavalent vaccine);confidence intervals (CI); ^+^Other includes: bednet distribution, vitamin A supplementation, deworming medication, told to return by healthcare worker, and unsure; +A child who received all antigens recommended by the Ghana Health Services including 1 dose of bacille Calmette–Guérin, 4 doses of oral polio vaccine, 3 doses of pentavalent (diphtheria-pertussis-tetanus-hepatitis B-*Haemophilus influenzae* type b) vaccine, 3 doses of pneumococcal conjugate vaccine, 2 doses of rotavirus vaccine, 2 doses of measles-containing vaccine, and 1 dose of yellow fever vaccine; ++A traditional method of communication during which the town crier beats a gong-gong, a metallic percussion instrument to draw people’s attention to messages he is providing; +++Answered by those with a child who stays with another adult during the day; ++++Answered by those with a child who attends a daycare; ^++^percentages account for clustering at the primary sampling unit (district) level and sampling weights.

#### Health facility organizational practices for immunization

Immunization health facility organizational practices are described in Table 4. Immunization defaulter tracing systems at health facilities were uncommon. In each region more than three-quarters of health facilities did not have a written list of immunization defaulters. Also, less than half of HCWs reported using a phone call to remind parents to return for immunizations (NR: 12%, VR: 44%, GAR: 47%). Communication between HCWs and supervisors over the phone was a more commonly reported practice. HCWs mainly called (NR: 87%, VR: 76%, GAR: 66%) and sent text messages (NR: 14%, VR: 36%, GAR: 26%) to communicate with their supervisors. The most common reasons for communicating with supervisors using a phone were to discuss immunization-related stock needs, to report vaccination data, and to discuss a specific immunization question.

**Table 4:** Health Facility (HF) organizational practices in three regions in Ghana, baseline survey, March 2016

	Northern RegionN = 52 HFs		Volta RegionN = 55 HFs		Greater Accra RegionN = 53 HFs	
	Number of responses	% (95%CI)^++^	Number of responses	% (95%CI)^++^	Number of responses	% (95%CI)^++^
**Written list of defaulters prepared**						
Yes, seen	0	0 (0-0)	5	9 (4-20)	2	4 (1-13)
Yes, not seen	3	6 (2-16)	8	15 (8-26)	6	11 (5-23)
No	49	94 (84-98)	42	76 (64-86)	45	85 (73-92)
**Phone or text messaging used to remind parents when to come for immunizations**						
Yes, phone calls	6	12 (5-23)	24	44 (31-57)	25	47 (34-60)
Both text messaging and phone calls or text messaging only	0	0 (0-0)	0	0 (0-0)	3	6 (2-15)
No	46	89 (77-95)	31	56 (43-67)	25	47 (34-60)
**Most common forms of communication with supervisor**						
Phone calls	45	87 (75-93	42	76 (64-86)	35	66 (53-77)
Text messaging	7	14 (7-25))	20	36 (25-50)	14	26 (16-40)
**Most common reasons for communicating with supervisor**						
Immunization-related stock needs	35	78 (64-88)	27	60 (46-73)	21	55 (40-70)
Immunization data reporting	12	27 (16-41)	12	27 (16-41)	12	32 (19-48)
Immunization specific questions	14	31 (20-46)	28	62 (48-75)	12	32 (19-48)
Impact of MCV2 introduction on health facility workload*	**N = 14**		**N = 25**		**N = 38**	
***The number of immunization sessions held (fixed or outreach)***						
Increased	10	71 (45-88)	14	56 (37-73)	28	74 (58-85)
Stayed the same	4	29 (12-55)	11	44 (27-63)	10	26 (15-42)
Decreased	0	0 (0-0)	0	0 (0-0)	0	0 (0-0)
***The time needed to hold an immunization session***						
Increased	9	64 (39-84)	16	64 (45-80)	21	55 (40-70)
Stayed the same	5	36 (16-61)	9	36 (20-56)	17	45 (30-60)
Decreased	0	0 (0-0)	0	0 (0-0)	0	0 (0-0)
***The time needed to document immunizations in the register***						
Increased	11	73 (48-89)	17	68 (48-83)	28	74 (58-85)
Stayed the same	4	27 (11-52)	7	28 (14-48)	10	26 (15-42)
Decreased	0	0 (0-0)	1	4 (1-20)	0	0 (0-0)

Abbreviations: second-dose measles-containing vaccine (MCV); confidence intervals (CI)

+ Among healthcare workers in the immunization workforce prior to 2012

++ percentages account for clustering at the primary sampling unit (district) level and sampling weights

To understand the impact of MCV2 introduction on HCW workload, staff who worked in immunization service delivery prior to MCV2 introduction in 2012 were asked additional questions (Table 4); these staff were at 14 health facilities in NR, 25 in VR, and 38 in GAR. Most staff interviewed indicated increases in the number of immunization sessions needed (NR: 71%, VR: 56%, GAR: 74%), the time needed to hold an immunization session (NR: 64%, VR: 64%, 55%), and the time required to document vaccinations in the register (NR: 73%, VR: 68%, GAR: 74%) due to the introduction of MCV2.

#### Healthcare worker practices

Of the HCWs selected for one-on-one interviews,72 were from participating health facilities in NR, 84 from VR, and 83 from GAR (Table 3). The job duties most commonly reported were administering vaccines (NR:89%, VR: 99%, GAR: 96%), health education and counseling (NR: 72%, VR: 84%, GAR: 82%), recording vaccinations in tally books (NR: 36%, VR: 58%, GAR: 65%), and registering children (NR: 50%, VR: 57%, GAR: 52%). Defaulter tracing was indicated by very few HCWs as one of their job duties (NR: 1%, VR: 2%, GAR: 1%). Most HCWs reported their last formal EPI training was more than a year ago (NR: 23%, VR: 28%, GAR: 26%) or unknown (NR: 59%, VR: 58%, GAR: 37%).

Table 5 describes individual HCW knowledge and practices. Approximately half of HCWs stated that immunizing both children under 12 months of age and older children was equally important (NR: 59%, VR: 54%, GAR: 54%). Catch-up immunization administration and documentation practices were assessed among HCWs through a series of questions asking the HCW what they would do in the scenario of an 18-month-old child who had not received MCV1. Most HCWs indicated correctly that they would give the child measles-rubella vaccine (MR), (NR: 59%, VR: 53%, GAR: 79%). A majority of HCWs in GAR, and lower proportions in other regions, reported correct immunization recording practices for a missed dose of MCV, recording as “MCV1 in the 12-23 month register” (NR: 38%, VR: 55%, GAR: 67%) and on the vaccination card as “9 Months, Measles 1”(NR: 54%, VR: 53%, GAR: 76%).Most (>78%) HCWs in each region correctly indicated they would tell the caregiver to return in one month for their child’s second MCV dose.

**Table 5:** Healthcare Worker (HCW) knowledge and practices among staff who provide immunization services in three regions in Ghana

	Northern RegionN = 72 HCWs		Volta RegionN = 84 HCWs		Greater Accra RegionN = 83 HCWs	
	Number of responses	% (95%CI)^++^	Number of responses	% (95%CI)^++^	Number of responses	% (95%CI)^++^
**In your opinion, is it more important to immunize infants who are under 12 months, children who are 12-23 months, or is the importance the same?**						
Same importance	40	59 (47-70)	45	54 (42-67)	45	54 (39-68)
More important to vaccinate infants < 12 months	31	40 (29-52)	38	45 (33-57)	36	44 (30-59)
More important to vaccinate children 12-23 months	1	1 (0-7)	1	1 (0-6)	2	2 (0-8)
**Scenario: An 18-month-old child arrives at the immunization session. He has not received ANY doses of measles-containing vaccine. *What vaccine do you administer during that visit?***						
Measles-rubella vaccine	42	59 (44-73)	44	53 (42-63)	63	79 (68-87)
Measles single antigen vaccine	29	39 (26-55)	40	48 (37-58)	19	20 (12-32)
Measles-containing vaccine (either of the above)	71	99 (93-100)	84	100 (-)	82	99 (94-100)
Do not offer vaccine	1	1 (0-7)	0	0 (0-0)	0	0 (0-0)
***How do you record this dose in the register?***						
As a first dose of measles immunization in the 0-11 month register	15	24 (14-39)	2	3 (1-11)	11	15 (8-28)
As a first dose of measles immunization in the 12-23 month register	27	38 (27-49)	47	55 (44-66)	55	67 (54-78)
As a second dose of measles immunization in the 12-23 month register	21	25 (16-38)	35	42 (31-53)	16	17 (10-28)
It is not recorded	3	6 (2 - 18)	0	0 (0-0)	0	0 (0-0)
Don’t know	5	7 (3 - 17)	0	0 (0-0)	0	0 (0-0)
***How do you record this dose on the Child Health Record book?***						
As a first dose of measles vaccine (“9 months, Measles 1”)	35	54 (42 - 66)	44	53 (42-63)	59	76 (64-85)
As a second dose of measles vaccine (“18 months, Measles 2”)	33	42 (30 - 54)	40	48 (37-58)	23	24 (15-36)
It is not recorded	0	0 (0-0)	0	0 (0-0)	0	0 (0-0)
Don’t know	3	5 (1 - 13)	0	0 (0-0)	0	0 (0-0)
***Tells caregiver to return in one month for second dose of measles-containing vaccine***						
yes	60	85 (75-92)	67	79 (67-87)	67	82 (69-90)

Abbreviations: confidence intervals (CI) **percentages account for clustering at the primary sampling unit (district) level and sampling weights

## Discussion

This study in three regions of Ghana provides insight into multiple challenges, both on the supply and demand sides, in achieving high vaccination coverage in the 2YL, potentially leaving many children under-protected from measles and other vaccine-preventable diseases (VPDs). Although all three regions achieved high (87%-99%) coverage and had relatively low (0%-11%) dropout rates for vaccinations delivered in the first year of life, coverage with MCV2, delivered in the 2YL, was modest (60%-83%) and dropout between MCV1 and MCV2 was substantial (14%-32%).

At the service delivery level, the ability of HCWs to identify children due for MCV2 is a critical factor for achieving and sustaining high coverage. Most (>75%) health facilities in our study lacked a systematic defaulter tracing system, and very few (<2%) HCWs indicated defaulter tracing as one of their job responsibilities. Guidance on a process for tracking children from the 9-month to the 18-month visit could help reduce drop-out and increase the proportion of children protected with two doses of MCV. Equipping HCWs with the appropriate recording tools for immunization services provided in the 2YL and providing guidance on how to use those tools is critical to developing effective defaulter tracing lists that reach beyond infancy.

Most HCWs had correct knowledge about administering a first dose of MCV to an older child, yet we observed inconsistencies on how the dose was recorded in the vaccination register and on the vaccination card. Vaccination cards used at the time of the survey had spaces for MCV labeled “9 Months, Measles 1” and “18 Months, Measles 2”, rather than labels indicating first dose and second dose. This labeling may have led HCWs to misinterpret the appropriate location to record the first dose of MCV if given at or around 18 months of age. Incorrect recording and reporting of MCV1 and MCV2 leads to inaccurate coverage estimates and potentially missed opportunities for vaccination among children who have not yet received two doses of vaccine.

Few HCWs reported receiving training on EPI topics within the last year or more, suggesting that much of the practical learning on immunization service delivery in the 2YL depends on peers. HCWs in GAR on average had more years of working experience, more recent exposure to EPI training, and were more likely to report correct vaccination recording practices than their counterparts in NR and VR. HCWs in all three regions reported an increase in their workload due to the introduction of MCV2, although both PCV and rotavirus vaccine were introduced in the same year so might have contributed to perceived increase in workload. Opportunities through in-service, new hire, and pre-service trainings can address knowledge and practice gaps among health workers needed to effectively administer vaccinations during the 2YL, and to develop effective strategies to manage the increased workload that immunization service delivery in the 2YL can bring.

A routine 2YL immunization visit is relatively new for most low- and middle-income countries and evidence for addressing challenges to improve coverage of vaccines administered in the 2YL is scarce. A recent Cochrane review sought to provide evidence for interventions aimed at improving child vaccination coverage in low- and middle-income countries including vaccines administered during the 2YL [9]. Findings highlighted the importance of improving service delivery by integrating immunization services with other health interventions, and improving access and demand by providing caregivers with information on immunization (both in their communities and during immunization visits), adequate reminders, and regular outreach services. In Ghana, as in many countries, a generation of parents has been accustomed to a single routine dose of measles vaccine. The addition of a second MCV dose requires a shift in messaging from public health authorities and behavior among caregivers. Despite social mobilization efforts during introduction, a high proportion of caregivers in all three survey regions were unaware of the need for MCV2, did not know the correct recommended ages for MCV, or did not perceive the vaccine as being of equal importance to vaccines given during the first year of life. Since most caregivers indicated HCWs as the most trusted source of information about immunization, HCWs have the opportunity and responsibility to communicate key messages about child health services administered in the 2YL and stress the importance of a child returning for additional immunizations in the childhood vaccination schedule. Our data show that HCWs missed opportunities to reinforce messages about MCV2 by not completing the return date on vaccination cards as a reminder to caregivers.

With the advent of mobile technology, more options are available for reinforcing immunization messages. Most caregivers reported they would accept information about childhood immunizations, including reminders for return visits, by text message, allowing for an additional point of communication and education between the HCW and caregiver. Although about half of HCWs reported calling caregivers to remind them to return for immunizations, only a small portion (<10%) utilized text messaging. Potential barriers for HCW to using mobile technology in communicating with caregivers, such as spotty cellular network, lack of caregiver access to a cellphone, and out-of-pocket costs associated with making calls need to be explored further in Ghana. In addition, caregiver demographic and cultural differences between regions should be considered when developing demand generation activities. For example in NR, most mothers had no or low education which may require a different approach to communication than that used for groups with higher education levels. Also, fewer caregivers in NR were receptive to text messages compared to caregivers in VR and GAR. In NR, combining traditional methods (e.g., gong-gong) with other methods of communication may be an effective approach for important messages about immunization.

In our study, up to one-third of 24-35 month old children were separated from their caregiver during the day and many of those children attended daycare, similar to national observations in Ghana [10]. In this context, access to immunization services may be logistically challenging. The success of school-based vaccination delivery programs in several countries has been well-documented; integrating immunization services into daycare centers could facilitate access to vaccination for daycare-attending children [11-14]. Among surveyed caregivers with children currently enrolled in daycare, most indicated they would allow their child to be given immunizations while in daycare. Daycare-based immunization services may help prevent VPD outbreaks among enrolled children, their families, and community contacts [15, 16].

There are several limitations to be considered when interpreting the results from this survey. First, data falsification was discovered in VR and GAR, leading to the exclusion of roughly 8% of sampled households. If some false data were undetected this would have reduced the precision of our estimates. However, the false data entry followed the child eligibility skip pattern that resulted in the questionnaire ending early, prior to caregiver responses about immunizations, so estimates presented here were not likely impacted. Second, the DHS sampling frame was from 2014 and roughly 20% of the sampled households were not found. This could have introduced selection bias if the households not found differ from the households that were interviewed. Vaccine coverage data relied on vaccination cards, which might have resulted in underestimating coverage since children without a card were assumed to be unvaccinated. Lastly, age discrepancies were identified when cross-validating variables; incorrect age could result in classification error for age-specific outcomes.

Strengthening the 2YL platform in Ghana will undoubtedly require a variety of interventions to address low MCV2 immunization coverage. Effective communication with caregivers, additional training and supportive supervision for the healthcare workforce, and system changes to improve defaulter tracking and data recording and reporting practices, will most likely be common needs across countries introducing a 2YL service delivery platform into their childhood immunization programs. Investment in this platform may provide benefits beyond the immunization system itself. Establishing or strengthening an interaction with the health system in the second year of life can improve trust between caregiver and the health system, reinforce health messages given during infancy, and provide new opportunities for health messages that are essential to fostering an environment for timely health interventions. Such interventions also augment global health security by facilitating disease detection and response efforts to prevent outbreaks before they start. Country experiences from the 2YL platform, lessons learned, and best practices should be documented and shared to provide guidance for future efforts globally.

## Conclusion

Immunization service delivery during the 2YL presents a unique set of challenges compared with immunizations administered during the first year of life. Strengthening a 2YL service delivery platform has numerous possible benefits and opportunities as it provides an additional contact for immunization to catch-up on missed doses and to integrate other child health interventions. A multi-faceted approach using a variety of interventions targeting HCWs, caregivers, and the health system itself might be an effective approach to achieve 2YL service delivery platform strengthening and ultimately help Ghana achieve high coverage for MCV2 and other vaccines scheduled for the 2YL.

### What is known about this topic

- Ghana has had relatively high vaccination coverage (≥ 85%) for infant antigens including a first dose measles-containing vaccine since 2007;
- In 2012, Ghana introduced a second dose measles-containing vaccine(MCV2), the first non-infant vaccine in the childhood immunization schedule, as a newly established 18-month visit simultaneously with pneumococcal conjugate vaccine (PCV) and rotavirus vaccine;
- MCV2 coverage has remained below 70% four years after introduction while coverage with the last doses of PCV and rotavirus vaccine had reached ≥ 89% during the same period.

### What this study adds

- Less than half of caregivers knew the recommended ages for children receiving vaccination against measles;
- Most health facilities lacked a systematic defaulter tracing system for identifying children due for MCV2, a critical factor for achieving and sustaining high coverage;
- More than half of caregivers indicated they were willing to accept text messages including reminders related to their child’s immunizations, however < 10% of health facilities were utilizing this practice.

## Competing interests

The authors declare no competing interest.
